# Efficacy and safety of CGRP monoclonal antibodies in chronic migraine: a systematic review integrating randomized and real-world evidence

**DOI:** 10.1007/s10072-026-09153-7

**Published:** 2026-06-09

**Authors:** Diamante Carta, Viviana Lo Buono, Rosario Grugno, Riccardo Lo Presti, Angelo Quartarone, Silvia Marino

**Affiliations:** IRCCS Centro Neurolesi “Bonino Pulejo”, S.S. 113 Via Palermo C/da Casazza, Messina, 98123 Italy

**Keywords:** Chronic migraine, CGRP monoclonal antibodies, Migraine prevention, Efficacy, Safety, Real-world evidence

## Abstract

**Background and objectives:**

Chronic migraine (CM) is a highly disabling neurological disorder characterized by ≥ 15 headache days per month, of which at least 8 exhibit migrainous features. Despite the availability of preventive therapies, conventional treatments are often limited by suboptimal efficacy and poor tolerability. Monoclonal antibodies targeting calcitonin gene–related peptide (CGRP) or its receptor have emerged as mechanism-based preventive options. This systematic review aims to evaluate the efficacy, safety, and clinical relevance of CGRP-targeted monoclonal antibodies in the prevention of chronic migraine.

**Materials and methods:**

A comprehensive literature search of PubMed, Scopus, and Web of Science was conducted from database inception to July 2025. Randomized controlled trials and observational real-world studies evaluating CGRP-targeted monoclonal antibodies in adult patients with chronic migraine were included. The review followed PRISMA 2020 guidelines (Page et al. 2021), was registered in the PROSPERO database (CRD420261284751), and included 11 studies out of 1,688 identified records.

**Results:**

Across included studies, CGRP-targeted monoclonal antibodies consistently demonstrated reductions in migraine frequency, improvements in ≥ 50% responder rates, and favorable safety profiles. Randomized controlled trials showed robust efficacy compared with placebo, while real-world studies confirmed effectiveness in more heterogeneous and treatment-resistant populations. However, variability in outcome definitions, particularly between monthly migraine days and monthly headache days, and differences in study design contributed to heterogeneity across findings.

**Conclusions:**

CGRP-targeted monoclonal antibodies represent an effective and well-tolerated preventive option for chronic migraine, with clinically meaningful benefits across both controlled and real-world settings. While current evidence is strong, further research is needed to evaluate long-term outcomes, optimize treatment strategies, and improve standardization of outcome measures.

**Trial registration:**

This systematic review was registered in the PROSPERO database (CRD420261284751).

**Supplementary Information:**

The online version contains supplementary material available at 10.1007/s10072-026-09153-7.

## Introduction

Chronic migraine (CM) represents a sustained disorder of the trigeminovascular system, characterized by persistent nociceptive sensitization rather than episodic attack recurrence alone. Clinically, it is defined by ≥ 15 headache days per month, of which at least 8 exhibit migrainous features, and is associated with a substantial burden in terms of quality of life, work productivity, and psychological well-being [1, 3].

Increasing evidence indicates that dysregulation of calcitonin gene–related peptide (CGRP) signaling plays a central role in maintaining this pathological state. Within this complex pathophysiological framework, CGRP has emerged as a key mediator linking peripheral trigeminal activation to central pain amplification. The recognition of CGRP as a pivotal driver of migraine pathogenesis has marked a paradigm shift in preventive treatment, enabling the development of mechanism-based therapies specifically targeting the CGRP pathway, including monoclonal antibodies directed against the CGRP ligand or its receptor [7, 10].

The clinical relevance of CGRP blockade was first established by randomized controlled trials of erenumab, which demonstrated significant preventive efficacy and favorable tolerability across migraine populations [20]. These findings were subsequently reinforced by international and European consensus statements and clinical guidelines, which now recognize CGRP-targeted therapies as evidence-based preventive options for both episodic and chronic migraine [18]. In addition, real-world studies have provided complementary evidence supporting their effectiveness in more heterogeneous and treatment-refractory populations [15].

Since the approval of erenumab, additional monoclonal antibodies targeting the CGRP ligand have expanded the therapeutic landscape. Galcanezumab and fremanezumab have shown consistent efficacy in patients with CM, including those with prior preventive treatment failures, as demonstrated in pivotal trials such as REGAIN, CONQUER, and FOCUS [5, 8, 11]. Eptinezumab, administered intravenously, has further highlighted the potential for rapid onset of preventive benefit, including in clinically challenging subgroups such as patients with medication-overuse headache, although heterogeneity in primary endpoint achievement has been observed across studies [24].

Despite the robustness of this evidence base, important clinical and methodological uncertainties remain. In particular, the optimal positioning of CGRP-targeted therapies within the preventive treatment pathway of CM—especially with respect to disease refractoriness, timing of initiation, and real-world effectiveness—has not been fully clarified. Moreover, existing evidence syntheses often pool heterogeneous endpoints, such as monthly migraine days (MMDs) and monthly headache days (MHDs), without adequately addressing their non-equivalence, and frequently report risk-of-bias assessments without fully integrating them into clinical interpretation. Limited distinction between randomized controlled trials and real-world evidence may further obscure differences in treatment response across clinical settings, particularly in treatment-resistant populations.

This review summarizes the available evidence on CGRP-targeted therapies for chronic migraine prevention. In contrast to previous reviews, we integrate data from randomized controlled trials and real-world studies across different migraine phenotypes and degrees of treatment refractoriness. Particular attention is given to distinguishing evidence derived from randomized and real-world settings, addressing endpoint heterogeneity through separate evaluation of MMDs and MHDs, and incorporating methodological quality into the interpretation of efficacy and safety outcomes, with the aim of providing a clinically oriented and conceptually integrated interpretation of current evidence.

## Materials and methods

This systematic review was conducted to assess the efficacy and safety of monoclonal antibodies targeting the CGRP pathway as preventive treatments for chronic migraine (CM). The review adhered to PRISMA 2020 guidelines [16] and was prospectively registered in the PROSPERO database (CRD420261284751).

A comprehensive search strategy was applied across three major biomedical databases (PubMed, Scopus, and Web of Science) to capture relevant literature. The search combined controlled vocabulary and free-text terms (e.g., “chronic migraine,” “CGRP,” “monoclonal antibodies,” “preventive treatment,” “efficacy,” “safety”), including specific drug names (erenumab, fremanezumab, galcanezumab, and eptinezumab), across all searchable fields to maximize sensitivity and scope. The predefined search strategy was adapted for each database. No restrictions on publication date were applied, and only studies published in English were considered.

**Fig. 1:**
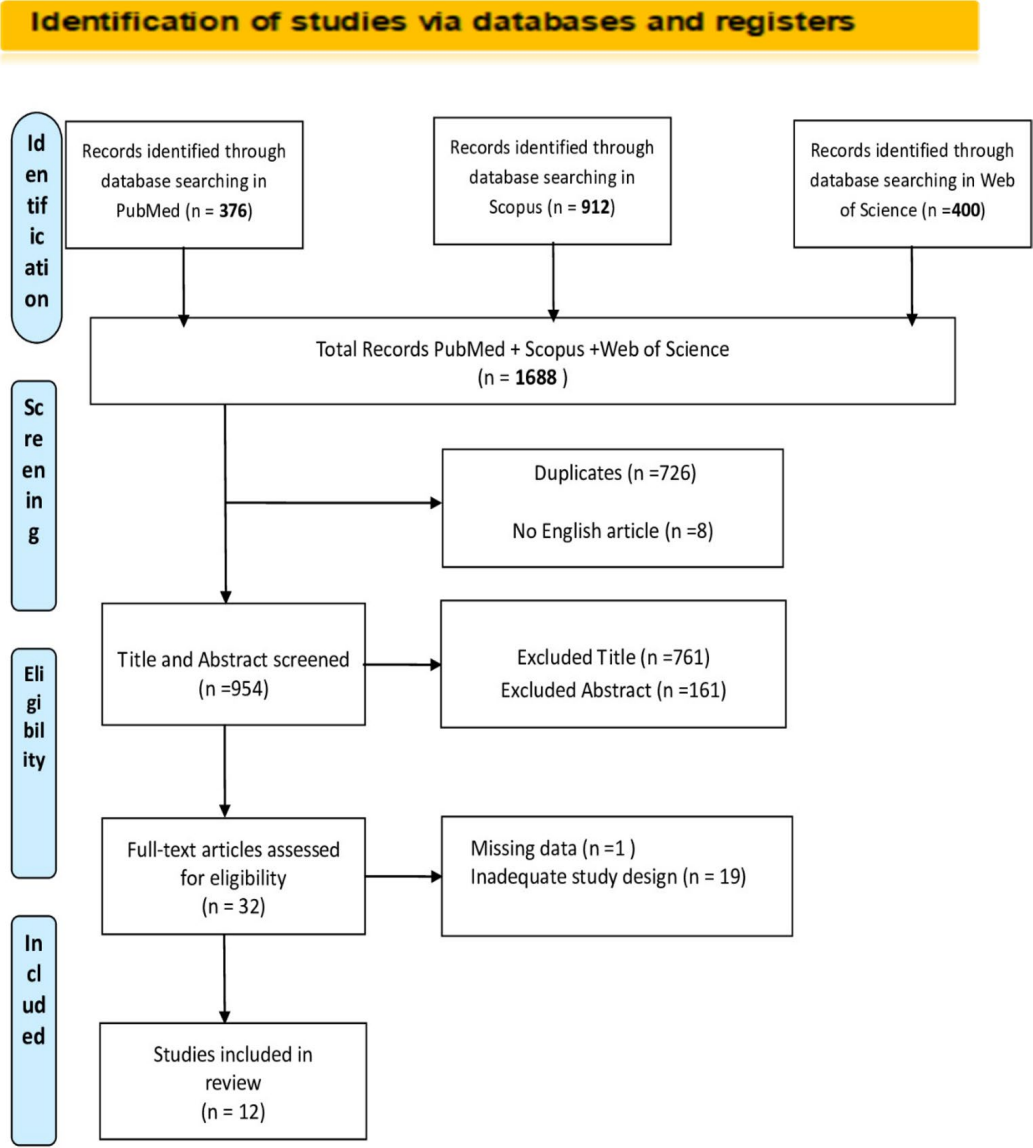
PRISMA flow diagram of study selection

The study selection process followed the PRISMA framework, including identification, screening, eligibility, and inclusion phases (see Fig. 1). The initial search yielded 1,688 records (PubMed: 376; Scopus: 912; Web of Science: 400). After removal of 726 duplicates, 954 records were screened by title and abstract. Of these, 922 were excluded for lack of relevance to CGRP-targeted prevention in CM. Thirty-two full-text articles were assessed for eligibility. Following full-text assessment, studies were excluded due to missing outcome data, non-comparable interventions, or inadequate methodological quality, resulting in 11 studies included in the final qualitative synthesis.

The research question and eligibility criteria were defined according to the PICO framework. The population consisted of adult patients (≥ 18 years) diagnosed with CM (≥ 15 headache days per month, of which at least 8 with migrainous features). The intervention included monoclonal antibodies targeting the CGRP pathway (erenumab, galcanezumab, fremanezumab, and eptinezumab). Comparators included placebo or standard oral preventive therapies when available. Outcomes were evaluated in terms of changes in monthly migraine days (MMDs) or monthly headache days (MHDs), ≥ 50% responder rates, and safety outcomes, including adverse events and treatment discontinuation, with particular attention to the distinction between MMDs and MHDs.

Eligible studies included randomized controlled trials and real-world observational studies conducted in human populations and published in English, provided they reported extractable efficacy and/or safety outcomes in CM populations or in mixed populations with relevant CM data. Systematic reviews, meta-analyses, editorials, conference abstracts, and non-English articles were excluded, although their reference lists were screened to identify additional relevant studies.

Data extraction was performed systematically to collect information on study design, patient characteristics, intervention details, efficacy outcomes, and safety profiles. Particular emphasis was placed on differences in outcome definitions, especially regarding MMDs and MHDs, to ensure appropriate interpretation of results.

The methodological quality of included studies was assessed using the Cochrane Risk of Bias 2 (RoB 2) tool for randomized controlled trials and the ROBINS-I tool for observational studies. Risk-of-bias assessments were incorporated into the interpretation of findings, particularly when comparing randomized and real-world evidence.

Given the heterogeneity across studies in terms of design, patient populations, follow-up duration, and outcome definitions (MMDs vs. MHDs), a quantitative meta-analysis was not performed. Instead, a qualitative synthesis was conducted. Randomized controlled trials and observational studies were analyzed separately, and outcomes based on MMDs and MHDs were evaluated independently to avoid inappropriate comparisons.

## Results

### Quality of included studies and risk of bias

Full-text evaluation according to the predefined eligibility criteria led to the inclusion of 11 studies, encompassing adult patients with chronic migraine (Fig. 1). All included studies evaluated the efficacy and safety of therapies targeting the CGRP pathway for preventive treatment. Across studies, primary efficacy outcomes were defined as reductions in monthly migraine days (MMDs) or monthly headache days (MHDs), depending on study design, and were compared with placebo or active comparators (see Table 1).

Risk of bias was assessed using validated methodological tools: the Revised Cochrane Risk of Bias Tool for Randomized Trials (RoB 2) for randomized controlled trials and the Risk of Bias in Non-randomized Studies of Interventions (ROBINS-I) for observational studies. Visual summaries were generated using the robvis tool.

Risk of bias was assessed separately for randomized controlled trials (RCTs) and observational studies. Among randomized controlled trials [5, 8, 11, 12, 17, 20, 23, 24], most were judged to be at overall low risk of bias, reflecting robust study design, appropriate randomization processes, and consistent outcome assessment. Some concerns were identified in specific domains, particularly related to deviations from intended interventions and missing outcome data [24].

**Fig. 2:**
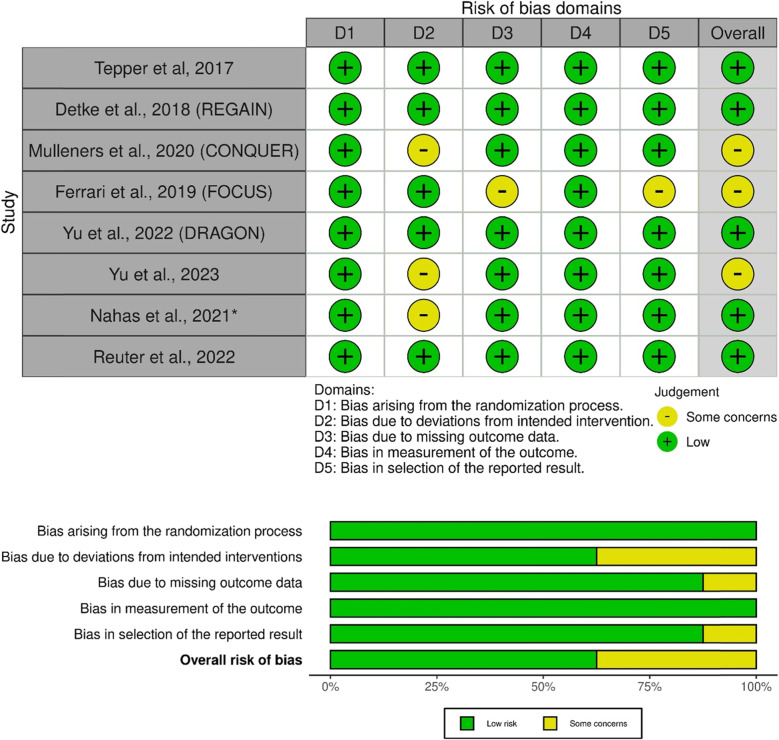
Study-level risk-of-bias assessment of randomized controlled trials (RoB 2)

**Fig. 3:**
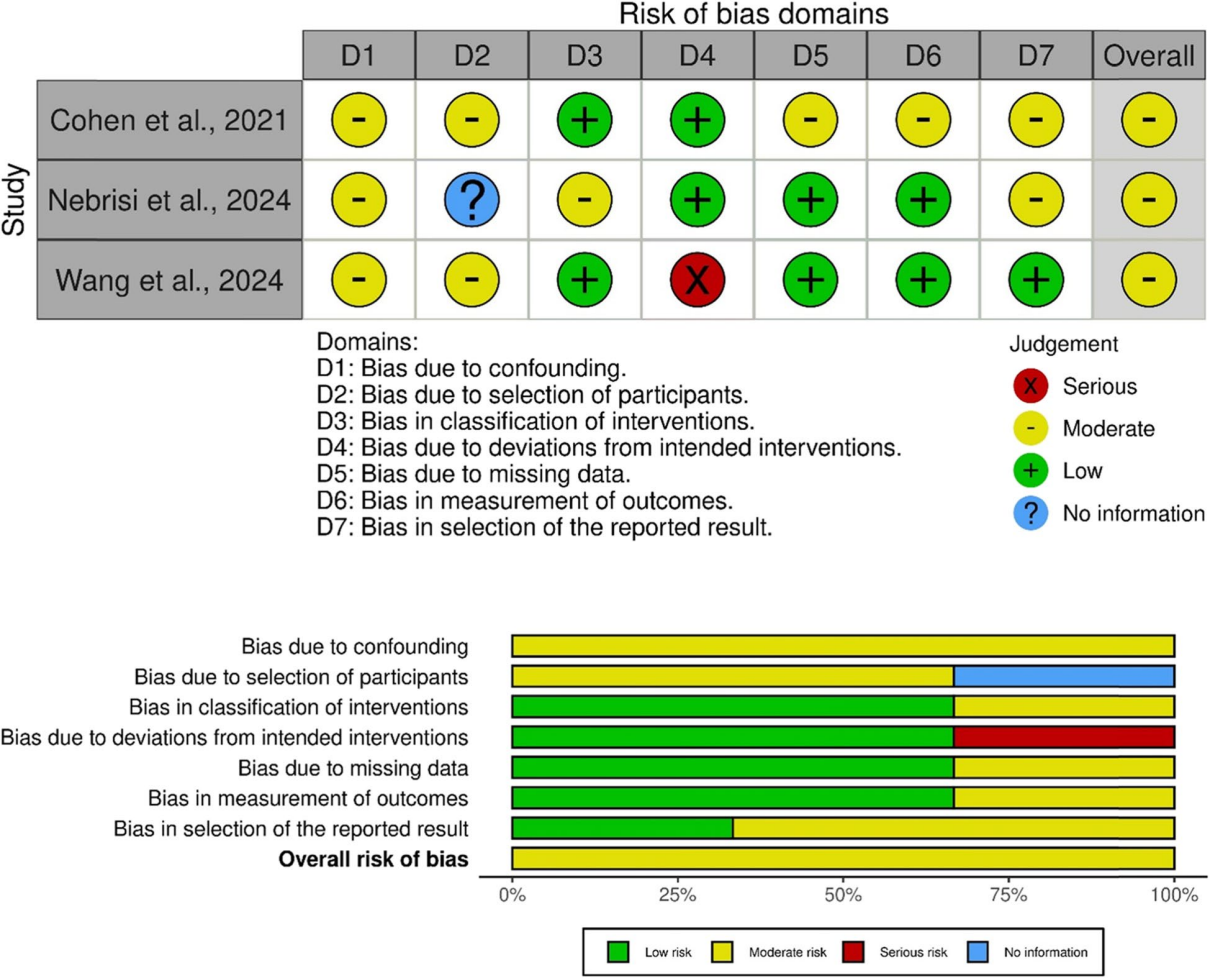
Study-level risk-of-bias assessment of non-randomized studies (ROBINS-I)

In contrast, observational studies [4, 13, 22] were judged to be at moderate to serious risk of bias, mainly due to confounding and participant selection, with additional concerns regarding non-random treatment allocation. Overall, these assessments indicate a robust randomized evidence base supporting CGRP-targeted therapies, while highlighting the need for cautious interpretation of observational findings. Risk-of-bias assessments were taken into account in the interpretation of results. Full study-level assessments are presented in Fig. 2 (RoB 2) and Fig. 3 (ROBINS-I).

Detailed study-level risk-of-bias assessments across individual domains for randomized and non-randomized studies are presented in Figs. 2 and 3, respectively.

## Study selection and characteristics

A total of 1,688 records were identified through database searching. After removal of duplicates (*n* = 726), 954 records underwent title and abstract screening, of which 922 were excluded. Thirty-two full-text articles were assessed for eligibility, and 21 were excluded due to inadequate study design or lack of relevant outcome data. Ultimately, 11 studies met the predefined inclusion criteria and were included in the qualitative synthesis (Figure X).

The included studies evaluated the efficacy and safety of CGRP-targeted monoclonal antibodies in patients with chronic migraine across both randomized and real-world settings. Overall, the body of evidence consistently demonstrated clinically meaningful reductions in migraine frequency, improved responder rates, and favorable safety profiles, although variability was observed depending on study design, patient characteristics, and outcome definitions.

## Evidence from randomized controlled trials

The pivotal phase 2 trial by Tepper et al. [20] provided the first strong evidence of preventive efficacy for erenumab. In this study, 667 patients were randomized to placebo or erenumab, showing a reduction of − 6.6 monthly migraine days compared with − 4.2 days in the placebo group.

Subsequent phase 3 trials confirmed and extended these findings. In the REGAIN study, galcanezumab significantly reduced monthly headache days compared with placebo (− 4.8 vs. − 2.7 MHD) [5]. Similarly, the CONQUER trial demonstrated that galcanezumab achieved clinically meaningful reductions in MMDs (− 4.1 vs. − 1.0) in patients with prior preventive treatment failures [11]. Fremanezumab showed comparable efficacy in treatment-resistant populations in the FOCUS trial (− 4.1 vs. − 0.6 MMD) [8].

Additional randomized evidence confirmed efficacy across different populations. The DRAGON study demonstrated significant reductions in MMDs in Asian patients treated with erenumab (− 8.2 vs. − 6.6) [23]. In contrast, the SUNLIGHT trial evaluating eptinezumab in patients with chronic migraine and medication-overuse headache did not meet its primary endpoint, although numerical improvements in secondary outcomes were observed [24]. Pooled analyses further supported the efficacy of fremanezumab in older populations [12].

Head-to-head evidence further supported the therapeutic profile of CGRP-targeted therapies. In the HER-MES trial, erenumab showed superior tolerability and treatment retention compared with topiramate, with markedly lower discontinuation rates due to adverse events (10.6% vs. 38.9%) [17].

Evidence from routine clinical practice further corroborated these findings. Real-world observational studies demonstrated consistent reductions in migraine frequency and clinically meaningful responder rates across different monoclonal antibodies. A multicenter cohort study showed greater reductions in MMDs with CGRP-targeted monoclonal antibodies compared with onabotulinumtoxinA (− 13.0 vs. − 8.7) [22]. Similarly, a retrospective cohort study reported higher response rates and better tolerability with erenumab compared with topiramate [13]. In addition, add-on therapy studies showed further reductions in monthly headache days when CGRP-targeted therapies were combined with onabotulinumtoxinA (− 5.7 MHD) [4].

A relevant source of variability across studies was the heterogeneity in outcome definitions, particularly the use of MMDs versus MHDs, which are not directly interchangeable. Randomized trials more frequently used MMDs, whereas some studies, such as REGAIN, reported MHDs as the primary endpoint, complicating direct comparisons across studies.

Across both randomized and observational studies, CGRP-targeted monoclonal antibodies demonstrated favorable safety and tolerability profiles. Adverse events were generally mild to moderate and comparable to placebo in randomized trials, while real-world studies confirmed low discontinuation rates and good tolerability.

Given the heterogeneity in safety reporting, including differences in adverse event definitions, reporting thresholds, outcome definitions (MMDs vs. MHDs) and follow-up duration, a quantitative pooled analysis of safety outcomes was not considered methodologically appropriate.

Overall, the available evidence demonstrates consistent and clinically meaningful reductions in migraine burden across CGRP-targeted monoclonal antibodies, supported by both randomized and real-world data, with favorable safety profiles.

The main characteristics and key findings of the included studies are summarized in Table 1, stratified by study design.

**Table 1:** Summary of included studies evaluating CGRP monoclonal antibodies in chronic migraine

Author (Year)	Study design	Population (*n*)	Intervention / Comparator	Primary outcome	Follow-up	Key results
*Randomized Controlled Trials*						
Tepper et al. [20]	Phase 2 RCT	667	Erenumab vs. placebo	MMD	Weeks 9–12	−6.6 vs. − 4.2 MMD
Detke et al. [5] (REGAIN)	Phase 3 RCT	1,113	Galcanezumab vs. placebo	MHD, ≥ 50% responders	3 months	−4.8 vs. − 2.7 MHD
Mulleners et al. [11] (CONQUER)	Phase 3b RCT	462	Galcanezumab vs. placebo	MMD, ≥ 50% responders	3 months	−4.1 vs. − 1.0 MMD
Ferrari et al. [8] (FOCUS)	Phase 3b RCT	838	Fremanezumab vs. placebo	MMD, ≥ 50% responders	12 weeks	−4.1 vs. − 0.6 MMD
Yu et al. [23] (DRAGON)	Phase 3 RCT	557	Erenumab vs. placebo	MMD, ≥ 50% responders	12 weeks	−8.2 vs. − 6.6 MMD
Yu et al. [24] (SUNLIGHT)	Phase 3 RCT	193	Eptinezumab vs. placebo	MMD, ≥ 50% responders	12 weeks	−7.2 vs. − 5.9 MMD (primary endpoint not met)
Nahas et al. [12]	Pooled analysis of 3 RCTs	246 (≥ 60 years)	Fremanezumab vs. placebo	MMD, ≥ 50% responders	12 weeks	−4.6 / −4.3 vs. − 2.3 MMD
Reuter et al. [17] (HER-MES)	Phase 4 head-to-head RCT	777	Erenumab vs. topiramate	MMD, discontinuation	24 weeks	Discontinuation: 10.6% vs. 38.9%
*Comparative and Real-World Studies*						
Nebrisi et al. [13]	Retrospective cohort	108	Erenumab vs. topiramate	≥ 50% response (MIDAS)	3–6 months	79% vs. 15% improvement
Wang et al. [22]	Multicenter real-world cohort	649	CGRP-mAbs vs. onabotulinumtoxinA	MMD, ≥ 50% responders	3 months	−13.0 vs. − 8.7 MMD
Cohen et al. [4]	Real-world add-on study	153	CGRP-mAbs + onabotulinumtoxinA	MHD	3 months	−5.7 MHD

*CGRP* Calcitonin gene–related peptide, *MMD* Monthly migraine days, *MHD* Monthly headache days, *MIDAS* Migraine Disability Assessment, *RCT* Randomized controlled trial

## Discussion

### Principal findings

This systematic review shows that therapies targeting the calcitonin gene–related peptide (CGRP) pathway have fundamentally reshaped the preventive treatment landscape for chronic migraine. Across randomized controlled trials and real-world observational studies, CGRP-targeted monoclonal antibodies consistently produced clinically meaningful reductions in monthly migraine days, with responder rates superior to placebo and, in comparative settings, to conventional oral preventive therapies [13, 17].

Importantly, these benefits were not confined to selected trial populations but extended to clinically complex and difficult-to-treat patients, including those with multiple prior preventive treatment failures. Evidence from phase 3 and phase 3b trials supports the robustness and durability of efficacy across the CGRP monoclonal antibody class, even in populations traditionally considered treatment-resistant [8, 11]. Taken together, these findings indicate that the therapeutic relevance of CGRP blockade extends beyond individual agents, reflecting a class-wide mechanism that remains effective across different stages of disease severity and treatment history.

### Biological rationale and comparison with existing literature

The clinical efficacy of CGRP-targeted therapies is supported by a well-established pathophysiological framework identifying CGRP as a key mediator in migraine, increasingly understood as a disorder of central sensory processing [10]. This biological rationale is further reinforced by experimental and translational evidence demonstrating the role of the trigeminovascular system and CGRP signaling in migraine generation [7].

Initial support for CGRP pathway inhibition in chronic migraine prevention emerged from early-phase randomized trials. The phase 2 study by Tepper et al. [20] provided the first robust evidence of efficacy, demonstrating significant reductions in monthly migraine days with a safety profile comparable to placebo. These findings were subsequently confirmed by pivotal phase 3 trials. In the REGAIN study, galcanezumab significantly reduced monthly headache days [5], while the CONQUER trial extended these observations to highly treatment-resistant populations [11]. Similarly, fremanezumab demonstrated efficacy in patients with prior preventive failures in the FOCUS trial [8].

Beyond individual trials, meta-analytic evidence confirms the superiority of CGRP-targeted monoclonal antibodies over placebo in reducing migraine frequency and improving responder rates, with favorable tolerability profiles [6, 9]. In addition, guideline-based evaluations support their use as evidence-based preventive therapies, particularly in patients who have not responded adequately to conventional treatments [18].

A major advance in the evidence base was provided by head-to-head and comparative studies. The HER-MES trial demonstrated superior tolerability and treatment retention of erenumab compared with topiramate [17], findings that were consistent with real-world evidence showing improved effectiveness and tolerability compared with conventional oral preventives [13].

### Clinical impact and real-world relevance

Real-world evidence further supports the external validity of these findings, demonstrating that the efficacy observed in randomized trials is largely maintained in routine clinical practice. Observational studies confirm sustained effectiveness and favorable tolerability across heterogeneous patient populations [15, 22].

In addition, combination approaches—such as the use of CGRP-targeted monoclonal antibodies as add-on therapy to onabotulinumtoxinA—suggest potential strategies for optimizing treatment in highly treatment-resistant patients [4]. These findings highlight the flexibility of CGRP-targeted therapies within personalized treatment strategies.

Beyond reductions in migraine frequency, CGRP-targeted therapies have demonstrated meaningful improvements in patient-reported outcomes, including quality of life, work productivity, and daily functioning [2, 3, 19]. These outcomes reflect the substantial burden of chronic migraine and underscore the broader clinical relevance of effective preventive treatment.

However, treatment response is not uniform across all patient groups. Variability in outcomes, particularly in more complex populations such as those with medication-overuse headache, suggests that clinical heterogeneity plays a significant role in determining treatment response [14]. This highlights the need for further research aimed at identifying predictors of response and refining individualized treatment approaches.

### Methodological considerations and limitations

Despite the consistency of the available evidence, several limitations should be acknowledged. First, substantial clinical and methodological heterogeneity was present across studies, particularly regarding patient populations, prior treatment exposure, and study design.

Second, heterogeneity in outcome definitions—specifically the use of monthly migraine days (MMDs) versus monthly headache days (MHDs)—represents a key methodological limitation. These endpoints are not directly interchangeable and may influence the interpretation and comparability of results across studies.

Third, although randomized controlled trials provide high internal validity, their generalizability may be limited by selected patient populations. Conversely, real-world studies offer greater external validity but are inherently subject to confounding and selection bias, consistent with the moderate to serious risk of bias identified in this review.

Finally, follow-up duration was relatively short in several studies, limiting conclusions regarding long-term efficacy and safety. Although emerging data suggest sustained benefits, further long-term studies are required.

### Therapeutic implications and future directions

A central question in chronic migraine management is not whether CGRP-targeted therapies are effective, but how best to position them within treatment algorithms. The convergence of evidence from randomized and real-world studies suggests that their favorable tolerability and treatment persistence may support earlier use in selected patients, particularly those with high disease burden or poor tolerability to conventional therapies [18, 21].

Overall, the accumulated evidence supports a shift toward more personalized and flexible treatment strategies. Future research should focus on long-term outcomes, comparative effectiveness between agents, and the identification of predictors of response to guide individualized treatment decisions. Addressing these gaps will be essential to optimize preventive strategies and improve long-term outcomes in chronic migraine.

## Conclusions and future directions

This systematic review highlights how therapies targeting the calcitonin gene–related peptide (CGRP) pathway have transformed the preventive management of chronic migraine, shifting from non-specific pharmacological approaches toward mechanism-based treatment strategies grounded in disease pathophysiology. Across randomized controlled trials and real-world studies, CGRP-targeted monoclonal antibodies consistently demonstrated clinically meaningful reductions in migraine frequency, improved responder rates, and favorable tolerability profiles.

Importantly, these benefits extend to clinically complex and treatment-resistant populations, addressing a major unmet need in patients who have not responded adequately to conventional preventive therapies. Evidence from real-world and observational studies further supports the external validity of these findings, confirming sustained effectiveness and acceptable tolerability in more heterogeneous clinical settings, although such data should be interpreted with caution due to inherent risks of bias.

Beyond reductions in migraine frequency, CGRP-targeted therapies have been associated with improvements in patient-reported outcomes, including quality of life, functional capacity, and work productivity, underscoring their broader clinical relevance.

Despite these advances, several challenges remain. Heterogeneity in outcome definitions, particularly between monthly migraine days (MMDs) and monthly headache days (MHDs), limited long-term data, and variability in individual treatment response highlight the need for more standardized methodologies and a deeper understanding of disease phenotypes.

Future research should focus on long-term effectiveness and safety, direct head-to-head comparisons, and the identification of predictive factors to guide personalized treatment strategies.

In conclusion, CGRP-targeted monoclonal antibodies represent a cornerstone of chronic migraine prevention. Their integration into clinical practice supports a more personalized and flexible approach to treatment and reflects a broader transition toward precision medicine in migraine care.

## Electronic supplementary material

Below is the link to the electronic supplementary material.

Supplementary Material 1 (DOCX 212 KB)

Supplementary Material 2 (DOCX 147 KB)

## Data Availability

All data generated or analyzed during this study are included in this published article. Additional information is available from the corresponding author upon reasonable request.

## Abbreviations

CGRP: Calcitonin gene-related peptide
CM: Chronic migraine
EHF: European Headache Federation
MHD: Monthly headache days
MIDAS: Migraine Disability Assessment
MMD: Monthly migraine days
MOH: Medication-overuse headache
PICO: Population, Intervention, Comparison, Outcome
PRISMA: Preferred Reporting Items for Systematic Reviews and Meta-Analyses
PROSPERO: International Prospective Register of Systematic Reviews
PROs: Patient-reported outcomes
RCT: Randomized controlled trial
RoB 2: Risk of Bias 2
ROBINS-I: Risk Of Bias In Non-randomized Studies of Interventions
