# Supplementary figures and images for: Efficacy and safety of CGRP monoclonal antibodies in chronic migraine: a systematic review integrating randomized and real-world evidence
Source: Neurol Sci. 2026 Jun 9;47(7):559. doi: 10.1007/s10072-026-09153-7 (PMC13249669; doi:10.1007/s10072-026-09153-7)

***Table S1.*** *Study-level RoB 2 judgments by domain for randomized controlled trials.*

| *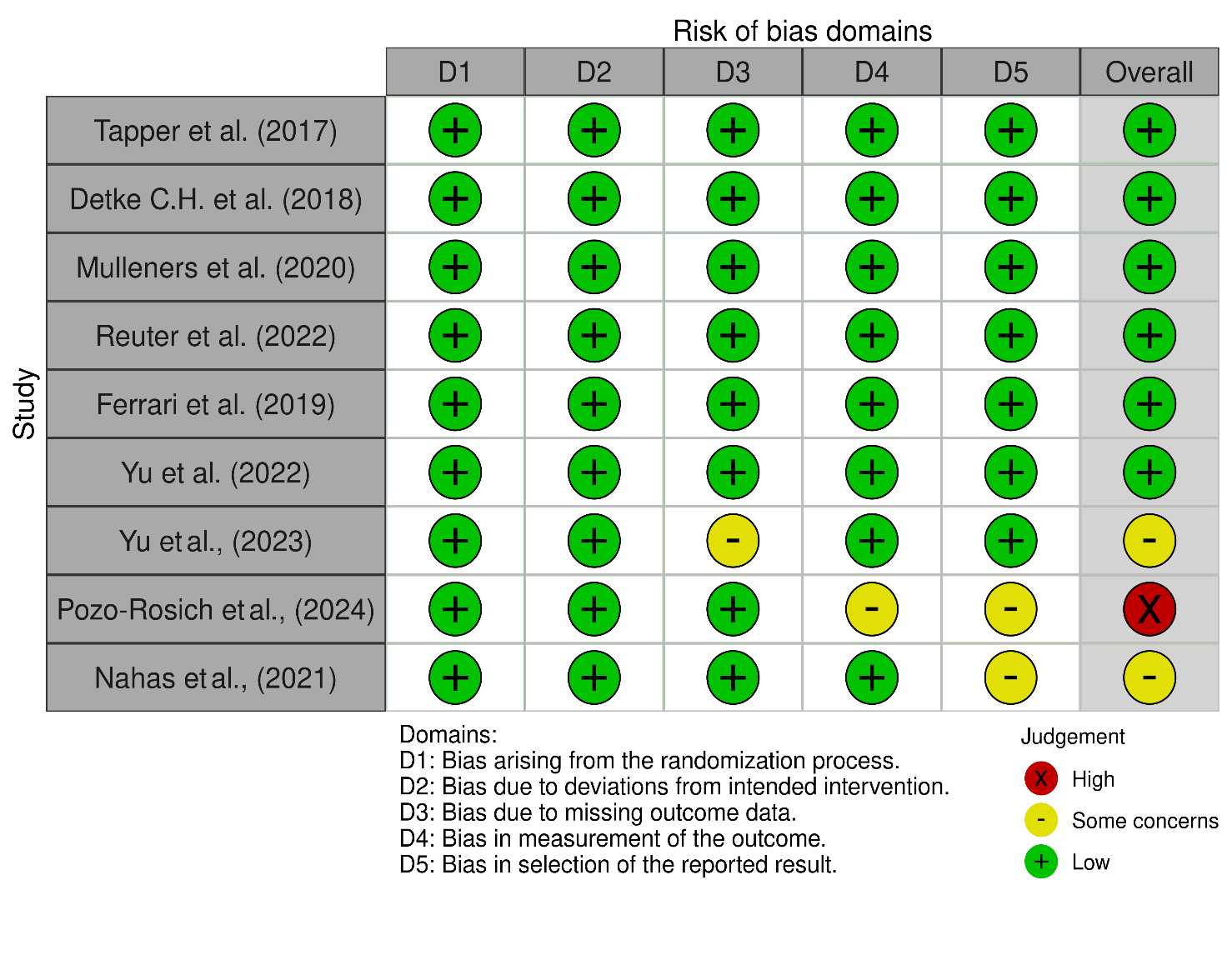* |
| --- |

Supplement: Supplementary file 1 — Supplementary Material 1 (DOCX 212 KB) [file 10072_2026_9153_MOESM1_ESM.docx]

***Table S2.*** *Study-level ROBINS-I judgments by domain for non-randomized studies.*

|  |
| --- |
| *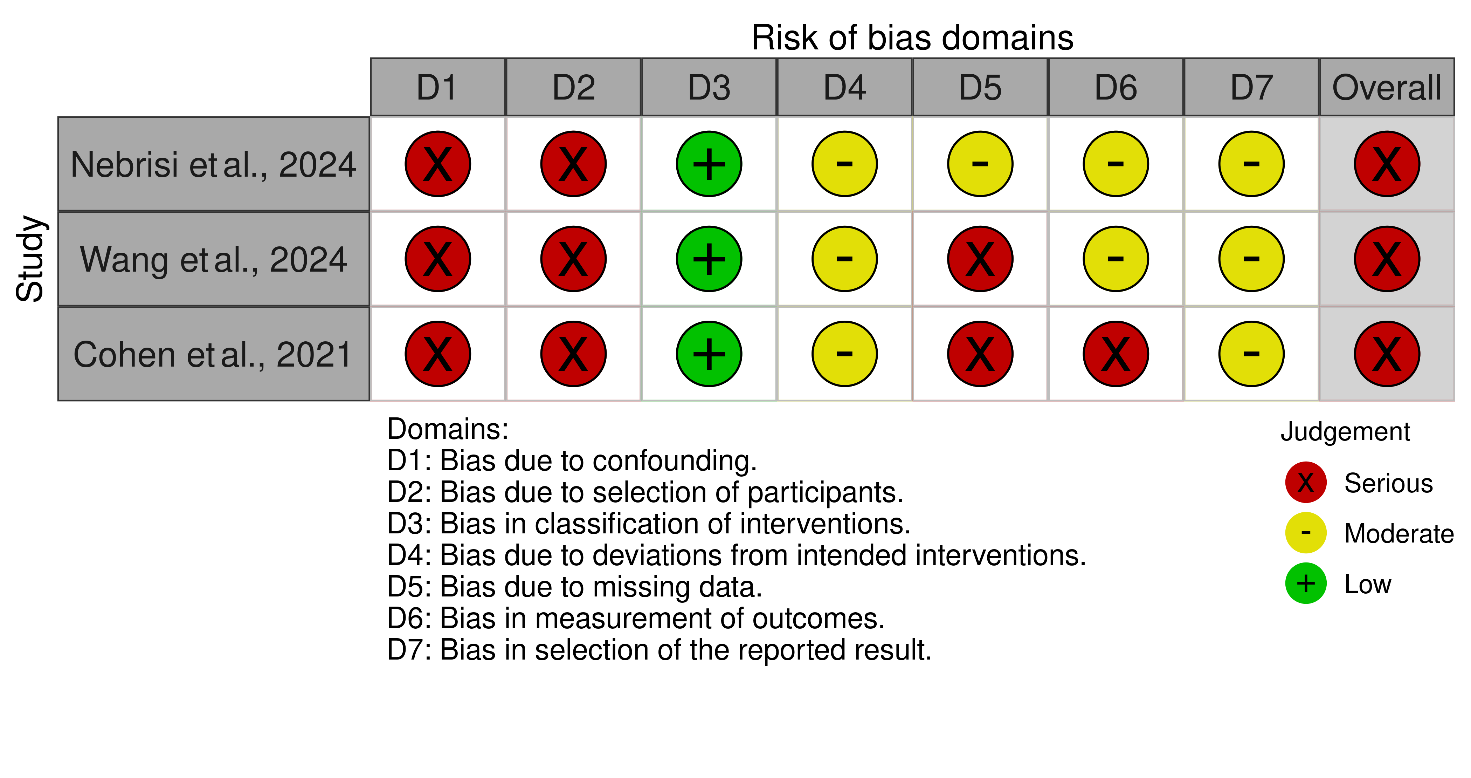* |

Supplement: Supplementary file 2 — Supplementary Material 2 (DOCX 147 KB) [file 10072_2026_9153_MOESM2_ESM.docx]
